# Impact of Angiography-Derived Physiological Patterns of CAD and Optimal Hemodynamics Post-PCI on Residual Angina

**DOI:** 10.1016/j.jacasi.2026.03.023

**Published:** 2026-05-16

**Authors:** Kotaro Miyashita, Yoshinobu Onuma, Emiliano Bianchini, Takashi Muramatsu, Gaku Nakazawa, Yuki Ishibashi, Ken Kozuma, Taku Asano, Yuki Katagiri, Takayuki Okamura, Yoshihiro Morino, Norihiro Kogame, Masafumi Ono, Yosuke Miyazaki, Shimpei Nakatani, Masato Nakamura, Akihiro Tobe, Asahi Oshima, Tsung-Ying Tsai, Scot Garg, Kengo Tanabe, Yukio Ozaki, John A. Spertus, Patrick W. Serruys

**Affiliations:** a Department of Cardiology, University of Galway, Galway, Ireland; b CORRIB Research Centre for Advanced Imaging and Core Lab, University of Galway, Galway, Ireland; c Department of Cardiology, Ageo Central General Hospital, Ageo, Japan; d Department of Cardiology, Fujita Health University Hospital, Toyoake, Japan; e Department of Cardiology, Kindai University, Osaka, Japan; f Department of Cardiology, School of Medicine, St. Marianna University, Kawasaki, Japan; g Department of Cardiology, Teikyo University Hospital, Tokyo, Japan; h Department of Cardiology, St. Luke International Hospital, Tokyo, Japan; i Department of Cardiology, Sapporo Higashi Tokushukai Hospital, Sapporo, Japan; j Division of Cardiology, Department of Medicine and Clinical Science, Graduate School of Medicine, Yamaguchi University, Yamaguchi, Japan; k Division of Cardiology, Iwate Medical University, Iwate, Japan; l Department of Cardiology, Tokyo Rosai Hospital, Tokyo, Japan; m Department of Cardiology, JCHO Hoshigaoka Medical Center, Osaka, Japan; n Division of Cardiovascular Medicine, Toho University Ohashi Medical Center, Tokyo, Japan; o Department of Cardiology, Royal Blackburn Hospital, Blackburn, United Kingdom; p School of Medicine, University of Central Lancashire, Preston, United Kingdom; q Division of Cardiology, Mitsui Memorial Hospital, Tokyo, Japan; r Department of Cardiology, Fujita Health University Okazaki Medical Center, Aichi, Japan; s University of Missouri-Kansas City’s Healthcare Institute for Innovations in Quality and Saint Luke’s Mid America Heart Institute, Kansas City, Missouri, USA

**Keywords:** completeness of revascularization, coronary artery disease, Murray law-based quantitative flow ratio, pullback pressure gradient index, quantitative flow ratio

## Abstract

**Background:**

Focal stenotic coronary lesions, as defined by the invasive pullback pressure gradient index (PPGI) measured before percutaneous coronary intervention (PCI), are associated with less residual angina than diffuse lesions as assessed by the Seattle Angina Questionnaire (SAQ).

**Objectives:**

This study aims to investigate the interaction between a lesion’s baseline functional phenotype (focal vs diffuse) and the physiological success of PCI, as defined by a post-procedural Murray-based angiography-derived flow ratio (μFR) above 0.90 (adequacy of flow [AOF]), in correlation with angina status at 2 years.

**Methods:**

The ASET-Japan study enrolled 203 patients with chronic coronary syndrome. The baseline functional disease pattern and the achievement of AOF (post-PCI μFR > 0.90) were analyzed as potential factors influencing the rate of residual angina at 2 years.

**Results:**

SAQ scores were obtained in 186 patients. The median follow-up duration was 771 days (Q1-Q3: 752-805). AOF was achieved in in 61.3% (114 of 186; 95% CI: 54.1-68.0), with the μFR post-PCI differing significantly by the lesion’s baseline phenotype (diffuse 0.92 ± 0.06 vs focal 0.94 ± 0.04; *P* < 0.001). At 2-year follow-up, patients in the AOF group with preprocedural focal vs diffuse disease had significantly better angina frequency scores (99.2 ± 3.3 vs 94.8 ± 11.7; *P* = 0.007), and less frequent residual angina (6.7%; 4 of 60; 95% CI: 2.6-16.2 vs 22.0%; 11 of 50; 95% CI: 12.5-34.9; *P* = 0.040).

**Conclusions:**

In patients who achieve AOF during PCI, the baseline functional phenotype of treated lesions in patients with or without angina at follow-up is significantly different. A global physiological assessment integrating AOF and the baseline functional pattern of disease is potentially useful to accurately predict residual angina post-PCI. (Acetyl Salicylic Elimination Trial Japan: The ASET Japan Pilot Study [ASET-JAPAN], NCT05117866)

The primary goal of mechanical revascularization in patients with chronic coronary syndrome (CCS) is to optimize their health status, symptoms, function, and quality of life by relieving or eliminating angina.^1^, ^2^, ^3^ However, after percutaneous coronary intervention (PCI), approximately a fifth of patients have residual or recurrent symptoms.^4^^,^^5^ In some, this may be due to the specific etiology of their angina (eg, microvascular disease or vasomotor disorders) whereas in others it reflects suboptimal post-PCI physiology. Recently, in a substudy of the TARGET FFR (Randomized Controlled Trial of Angiography Versus Pressure Ratio-Guided Enhancement Techniques—Fractional Flow Reserve) trial, Collet et al^6^ showed that the baseline functional pattern of coronary artery disease (CAD) (specifically diffuse or focal) was significantly associated with the degree of angina relief after PCI. Although this initial work showed that PCI of physiologically focal, as opposed to diffuse, coronary lesions resulted in less angina, it has been hypothesized that the strength of this association depends on the physiological success of PCI.

The potential interaction between the baseline functional pattern of CAD and a structured assessment of anginal symptoms, such as that provided by the Seattle Angina Questionnaire (SAQ) after PCI, has not yet been fully investigated with angiography-derived fractional flow reserve (FFR). Furthermore, previous studies did not investigate the baseline pattern of disease in the context of the completeness of revascularization or optimal functional revascularization post-PCI.^6^, ^7^, ^8^

The concept of adequacy of flow (AOF) after PCI, defined as an optimal functional result in treated vessels (FFR >0.90) combined with the absence of functionally significant untreated lesions (all untreated lesions with FFR >0.80), has been associated with the best clinical outcomes, mainly driven by lower repeat revascularization.^9^, ^10^, ^11^ The challenge of performing such comprehensive assessments with wire-based technology has recently been overcome by advances in angiography-based physiology software, which have made it possible to evaluate multiple functional parameters, such as baseline quantitative flow ratio and the pressure pullback gradient index (PPGI), in all 3 major coronary arteries using a single angiographic acquisition and without the need for dedicated pressure wires.^8^^,^^12^, ^13^, ^14^

To address the impact and interaction of lesion physiology at baseline and post-PCI, the ASET-Japan (Acetyl Salicylic Elimination Trial Japan (ASET-Japan; NCT05117866) Chronic Coronary Syndrome study (ASET-Japan CCS) was leveraged to investigate the baseline angiography-derived functional pattern of CAD in stenotic lesions and assess its interaction with the global AOF and patients’ angina status at 2 years as evaluated by the SAQ.

## Methods

### Study Design and Population

The current study is a subanalysis of the ASET-Japan pilot study. The design, methodology, and preliminary results of the study have already been published elsewhere.^15^^,^^16^ In brief, ASET-Japan was a first-in-humans, multicenter, single-arm, open-label trial that enrolled CCS patients with anatomical SYNTAX (Synergy Between PCI With Taxus and Cardiac Surgery) scores <23 who were undergoing PCI with the implantation of SYNERGY platinum-chromium Everolimus-eluting stents.^15^^,^^16^ The study was approved by the certified review board (CRB4180003) at Fujita Health University (Toyoake, Japan) and the local ethics committees at each investigating center, and all patients provided their written informed consent before participation in the study. Patients who died or were lost to follow-up and those who had no available SAQ at 2 years were excluded. Patients undergoing repeat revascularization (PCI and/or coronary artery bypass grafting) within 2 years were also excluded from these analyses because the additional intervention would be expected to alter the coronary physiology after the initial PCI.

### Analysis of Murray’s Law–Based Quantitative Flow Ratio and Functional Pattern of the Disease

Murray’s law–based quantitative flow ratio (μFR) was performed by an independent academic core laboratory (CORRIB Lab, University of Galway, Ireland) using the AngioPlus system (Pulse Medical Imaging Technology). The μFR is a computational method applied to a single angiographic view that considers side branch diameters in the computation of fractal flow division; it has recently been shown to have similar diagnostic accuracy to 3-dimensional quantitative flow ratio and a correlation of *r* = 0.90 (*P* <0.001) and acceptable agreement with wire-based FFR.^17^ The functional significance of stenotic lesions, the residual functional SYNTAX score, and optimal hemodynamics after PCI were assessed using μFR. The preprocedural functional distribution of plaque (focal vs diffuse) in treated lesions was assessed using the μFR-derived PPGI, calculated as follows:^6^^,^^7^^,^^18^

$$\text{PPG}\;\text{index}=\frac{\left\{\frac{\mathrm{Max}\;\text{PPG}\;20\;\text{mm}}{\Delta \mu \text{FR}\;\text{vessel}}+\left(1-\frac{\text{Length}\;\text{with}\;\text{functional}\;\text{disease}\;\left[\text{mm}\right]}{\text{Total}\;\text{vessel}\;\text{length}\;\left[\text{mm}\right]}\right)\right\}\;}{2}$$

Maximal PPGI was defined as the maximum μFR gradient over 20 mm, and ΔμFR vessel as the difference between μFR values obtained at the ostium of the vessel and the most distal point. The length with functional disease was defined as the length in millimeters, with a μFR drop ≥0.0015/mm.^13^ The total vessel length was defined as the length of the entire interrogated vessel. The physiological distribution of CAD was defined as predominantly diffuse or focal according to whether the pre-PCI μFR-derived PPGI was above (focal) or below (diffuse) the 0.68 median in our cohort.^7^^,^^19^

The μFR was computed for all vessels with a diameter of 1.5 mm or greater, as visually assessed by coronary angiography. The baseline functional assessment encompassed vessels that underwent PCI (treated vessels) and those that did not. The pre-PCI μFR-derived PPGI was only calculated in vessels in which PCI was performed during the index procedure. If more than 1 vessel was treated (n = 5), PPGI and lesion length were derived from the vessel with the lowest PPGI, and focal or diffuse phenotype was categorized accordingly.

### Core Laboratory Postprocedural Angiographic and μFR Analyses

#### Before PCI

In this study, the intention-to-treat decision was initially made by the site investigators based on their local assessment using visual quantitative diameter stenosis or a physiological assessment (Supplemental Figure 1). This decision was categorized as the “investigator-intended decision to treat.” Retrospectively, an independent core laboratory reassessed all visible (30%-50%) lesions and analyzed the baseline diameter stenosis using quantitative coronary angiography (QCA) and assessed functional significance using μFR (μFR ≤0.80 or >0.80). A retrospective “CoreLab intention-to-treat” decision was subsequently generated with vessels having a μFR ≤0.80 defined as “to be treated” regardless of their QCA results whereas those with μFR >0.80 were defined as “not to be treated” (Supplemental Figure 1).

#### After PCI

The CoreLab also retrospectively analyzed post-PCI angiograms to assess diameter stenosis using QCA, vessel μFR, and the intrastent ΔμFR. In this retrospective analysis, device success was defined according to the European Association of Percutaneous Cardiovascular Interventions recommendations,^20^ which were enhanced by the addition of a functional component consisting of an intrastent ΔμFR <0.05. According to this analysis, 144 patients achieved a post-PCI device success (QCA diameter stenosis <20% and intrastent ΔμFR <0.05). This retrospective post-PCI assessment of device success by the core lab is more quantitative and objective than the on-site assessment of successful implantation, which was a mandatory criterion for enrollment into the ASET trial.^15^ Furthermore, an optimal functional result requires a vessel μFR >0.90 in all treated lesions. Finally, global adequacy of flow (AOF) was defined as all treated lesions having an intrastent ΔμFR <0.05 and all untreated vessels having a μFR >0.80, which was considered to be functionally optimal when the post-PCI vessel had μFR >0.90.

The functional SYNTAX score before PCI and the residual functional SYNTAX score (SS) post-PCI^11^ were assessed by removing the weighting factor attributed to each coronary segment of the Leaman/SYNTAX segmentation whenever the segment had a μFR >0.80.^21^ Completeness of functional revascularization corresponding to a residual functional SYNTAX score of 0 was assessed based on post-PCI μFR values in “treated” vessels.

An angio-derived μFR <0.90 after PCI has a negative impact on major cardiovascular and cerebrovascular events.^1^^,^^21^^,^^22^ Post-PCI AOF can only be reasonably guaranteed if the μFR is >0.90 in treated vessels and >0.80 in untreated vessels; in other words, a post-PCI residual functional SYNTAX score of 0 does not fully guarantee being free from angina. Therefore, in the current study AOF was defined as completeness of functional PCI revascularization (residual functional SYNTAX score = 0) with an optimal post-PCI vessel μFR value of >0.90 in all treated vessels, in conjunction with a vessel μFR of >0.80 in all remaining untreated lesions. Patients who did not fulfil these criteria were categorized as being non-AOF patients (post-PCI vessel μFR <0.90 in any treated vessel and/or pre-PCI vessel μFR ≤ 0.80 in any nontreated vessel).

### Angina and Quality of Life Assessment by Seattle Angina Questionnaire

The 19-item Seattle Angina Questionnaire (SAQ-19) was filled out by patients 2 years after their procedure. The SAQ measures 5 domains related to angina: Angina Frequency, Physical Limitations, Quality of Life, Angina Stability, and Treatment Satisfaction. Scores can range from 0 to 100, with 0 signifying the worst possible health status and 100 indicating the best possible health status. An SAQ Summary score was calculated by averaging the scores from the SAQ Physical Limitations, SAQ Quality of Life, and SAQ Angina Frequency domains. Additionally, the presence of residual angina was explicitly defined as an SAQ Angina Frequency score <100, and angina-free status was defined as an SAQ Angina Frequency score of 100. SAQ Angina Frequency score was also categorized into 3 clinically relevant strata: daily/weekly angina (<60), monthly angina (60-99), and no angina (= 100). Questionnaire collection was conducted by a research nurse or cardiologist familiar with the SAQ.^23^

### Statistical Analysis

The SAQ Summary and the subdomain scores were calculated using the official SAS code provided by cvoutcomes.org (Saint Luke’s Mid America Heart Institute). Continuous variables were first tested for normality with the Shapiro-Wilk test. Normally distributed variables are presented as mean ± SD and were compared by Student’s *t*-test, whereas non-normal variables are given as median (Q1-Q3) and were compared using the Mann-Whitney *U* test. Categorical variables are expressed as counts and percentages and were compared by the chi-square test or Fisher’s exact test, as appropriate.

The SAQ summary score and subscores from its component domains were used as continuous variables and compared between patients with diffuse and focal CAD. Distribution curves were created using continuous values of each SAQ score, which were then compared between patients with focal and diffuse patterns of CAD in the overall cohort and in the AOF and non-AOF subgroups. Logistic regression analyses were performed to identify independent predictors of angina-free status at 2 years. Physiological disease pattern was primarily defined using the cohort median PPGI, consistent with prior invasive physiology studies. Sensitivity analyses were performed by treating PPGI as a continuous variable without dichotomization and by applying alternative percentile-based cut points (33rd percentile) (Supplemental Methods). Odds ratios were reported with 95% confidence intervals, and statistical significance was defined as a 2-sided *P* value < 0.05. A 2-sided *P* < 0.05 was considered statistically significant. All statistical analyses were performed using R version 4.1.3 (R Foundation for Statistical Computing).

## Results

A total of 206 patients were initially enrolled in the ASET-Japan CCS study. At 2-year follow-up, 3 patients had died (vascular death: n = 1, noncardiac death: n = 2). Seventeen of the remaining 203 patients who were alive at 2 years were excluded, 9 because of missing 2-year SAQ scores and 8 because they had undergone repeat revascularization. Therefore, 186 patients were included in the present study; however, a pre-PCI PPGI analysis was not possible in the 9 patients having a total occlusion, leaving 177 patients who had both a SAQ score and PPGI. The median follow-up duration was 771 days (Q1-Q3: 752-805). The Central Illustration summarizes the conceptual framework of this study, highlighting the interaction between baseline physiological phenotype, post-PCI adequacy of flow, and long-term angina outcomes.

**Central Illustration:**
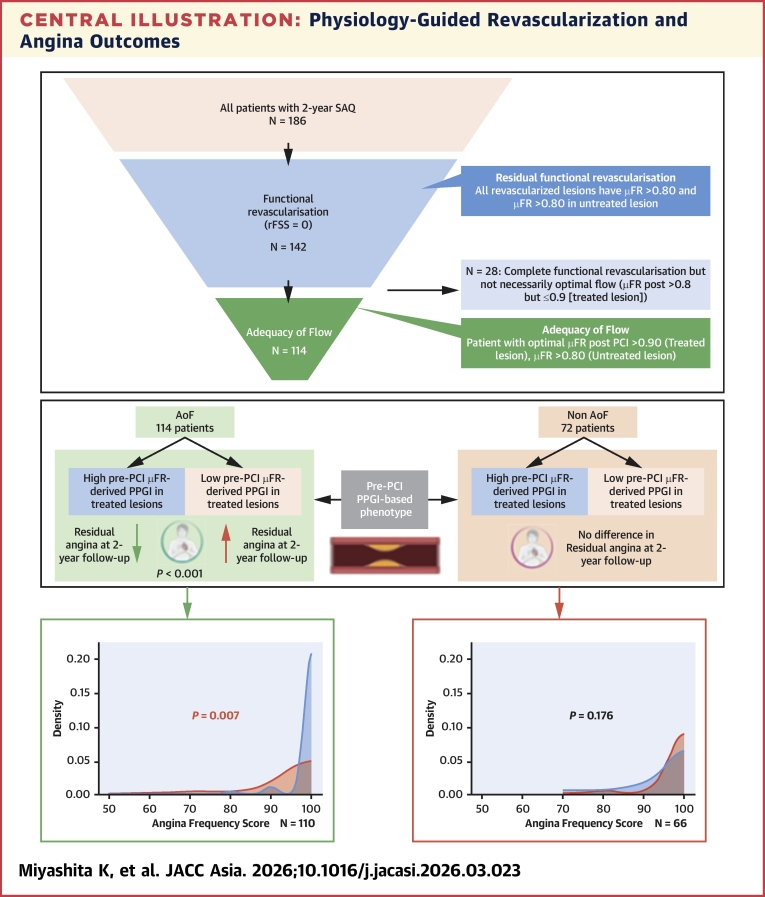
Physiology-Guided Revascularization and Angina Outcomes The central illustration shows the relationship between angiography-derived physiological disease pattern, adequacy of flow, and residual angina after percutaneous coronary intervention. Among patients achieving adequacy of flow, higher pre-percutaneous coronary intervention pressure pullback gradient index in treated vessels was associated with lower residual angina at 2 years. In contrast, no such association was observed in patients without adequacy of flow. These findings highlight that the prognostic relevance of angiography-derived physiology depends on the achievement of adequate post-PCI flow. AoF = adequacy of flow; percutaneous coronary intervention; μFR = Murray’s law-based quantitative flow ratio; PCI = percutaneous coronary intervention; PPGI = pressure pullback gradient index; rFSS = residual functional SYNTAX score; SAQ = Seattle Angina Questionnaire.

### Baseline Characteristics

Baseline clinical characteristics are detailed in Table 1. A total of 186 patients (96% of the entire trial cohort) were assessed with SAQ at follow-up and were included in the analysis. The mean age was 68.9 years old, and 79.4% were male. The median anatomical SYNTAX score was 7.0 (Q1-Q3: 5.0-11.0). Among these patients (N = 186), 144 achieved device success, and 142 had complete functional revascularization (residual functional SYNTAX score = 0). AOF was achieved in 61.3% (114 of 186; 95% CI: 54.1-68.0), and 38.7% (72 of 186) were classified as non-AOF. No significant differences were observed between the AOF and non-AOF groups in terms of patient characteristics, mean left ventricular ejection fraction, baseline anatomical SYNTAX score, or functional SYNTAX score (Table 1). In univariate logistic regression analyses, in the patients achieving AOF, the diffuse phenotype was a significant protector (OR: 0.234; 95% CI: 0.078-0.702; *P* = 0.010) against no-anginal status at 2 years. In patients without AOF, the diffuse phenotype had an increased odds (OR: 2.400; 95% CI: 0.584-9.856; *P* = 0.224) for nonanginal status although this was not statistically significant.

**Table 1:** Baseline Patient Characteristics

	Overall (N = 186)	AOF Group (n = 114)	Non-AOF Group (n = 72)	*P* Value
Age, y	68.90 ± 10.04	68.90 ± 9.92	68.89 ± 10.29	0.992
Male	148 (79.6)	86 (75.4)	62 (86.1)	0.116
Female	38 (20.4)	28 (24.6)	10 (13.9)	0.116
Body mass index, kg/m^2^	24.60 ± 3.81	24.25 ± 3.77	25.15 ± 3.83	0.118
Current smoking	33 (17.7)	19 (16.7)	14 (19.4)	0.775
Diabetes mellitus	65 (34.9)	37 (32.5)	28 (38.9)	0.460
Insulin-dependent	12 (6.5)	4 (3.5)	8 (11.1)	0.080
Hypertension	148 (79.6)	89 (78.1)	59 (81.9)	0.652
Dyslipidemia	157 (84.4)	91 (79.8)	66 (91.7)	0.050
Family history of CAD^a^	9 (4.8)	8 (7.0)	1 (1.4)	0.164
Established PVD	12 (6.5)	9 (7.9)	3 (4.2)	0.483
COPD	9 (4.8)	5 (4.4)	4 (5.6)	0.991
History of heart failure	13 (7.0)	10 (8.8)	3 (4.2)	0.366
History of major bleeding^b^	4 (2.2)	1 (0.9)	3 (4.2)	0.323
Renal insufficiency^c^	17 (9.1)	11 (9.6)	6 (8.3)	0.966
Previous PCI	45 (24.2)	28 (24.6)	17 (23.6)	1.000
Previous MI	24 (12.9)	17 (14.9)	7 (9.7)	0.421
Previous CABG	3 (1.6)	2 (1.8)	1 (1.4)	1.000
LVEF, %	60.9 ± 9.5	61.2 ± 9.6	60.5 ± 9.5	0.279
SYNTAX score				
Anatomical	7.0 (5.0-11.0)	7.0 (5.0-11.0)	7.0 (5.0-11.0)	0.282
Functional	6.0 (5.0-8.0)	6.0 (3.0-7.0)	6.0 (5.0-9.0)	0.203
CCS classification				0.653
I	61 (32.8)	39 (34.2)	22 (30.6)	
II	81 (43.5)	50 (43.9)	38 (50.0)	
III	8 (4.3)	5 (4.4)	3 (4.2)	
IV	2 (1.1)	2 (1.8)	0 (0)	
Silent ischemia	34 (18.3)	18 (15.8)	16 (22.2)	

Values are n (%), mean ± SD, or median (Q1-Q3).

AOF, adequacy of flow; CAD = coronary artery disease; CABG = coronary artery bypass graft; CCS, Canadian Cardiovascular Society; COPD = chronic obstructive pulmonary disease; LVEF = left ventricular ejection fraction; MI = myocardial infarction; PCI = percutaneous coronary intervention; PVD = peripheral vascular disease; SYNTAX = Synergy Between PCI With Taxus and Cardiac Surgery.

a History of coronary artery disease in first-degree relative.

b History of bleeding events requiring hospitalization within 1 year.

c Renal insufficiency is defined as an estimated glomerular filtration rate of creatinine clearance <60 mL/min/1.73 m^2^.

### Baseline and Retrospective Post-PCI Functional Characteristics and SAQ in AOF and Non-AOF Groups

Table 2 presents the functional characteristics and outcomes of patients with or without AOF after PCI, along with angina status stratified by lesion phenotype (diffuse vs focal). Data are presented for the overall cohort and subgroups based on the achievement of AOF. Additional comparisons of SAQ scores between AoF and non-AoF groups are provided in Supplemental Table 1. The median PPGI used to classify patients with focal or diffuse disease in this cohort was 0.68. In the overall cohort, compared with the focal disease group, the diffuse group had significantly longer lesion length (41 ±11 vs 22 ± 8 mm; *P* < 0.001) and a significantly lower post-PCI vessel μFR (0.92 ± 0.06 vs 0.94 ± 0.04; *P* = 0.009). The SAQ scores were similar between diffuse and focal groups in the overall cohort and in patients in the non-AOF subgroup.

**Table 2:** Functional Characteristics and Outcomes of Patients With or Without Adequacy of Flow

	Patients With PPGI and SAQ (N = 177)			AOF Group (N = 111)			Non-AOF Group (N = 66)		
	Diffuse (n = 88)	Focal (n = 89)	*P* Value	Diffuse (n = 51)	Focal (n = 60)	*P* Value	Diffuse (n = 37)	Focal (n = 29)	*P* Value
Preprocedure									
PPGI	0.57 ± 0.08	0.79 ± 0.07	<0.001	0.57 ± 0.09	0.80 ± 0.07	<0.001	0.57 ± 0.07	0.78 ± 0.07	<0.001
μFR	0.68 ± 0.13	0.65 ± 0.17	0.203	0.70 ± 0.11	0.67 ± 0.16	0.183	0.66 ± 0.14	0.63 ± 0.20	0.508
Lesion length, mm	41 ± 11.3	22 ± 8.1	<0.001	40 ± 11.7	22 ± 7.9	<0.001	41 ± 10.9	23 ± 8.5	<0.001
Postprocedure									
μFR	0.92 ± 0.06	0.94 ± 0.04	0.009	0.94 ± 0.04	0.95 ± 0.02	0.102	0.89 ± 0.07	0.91 ± 0.05	0.090
ΔμFR across the stent	0.01 (0.00-0.01)	0.01 (0.00-0.01)	0.279	0.01 (0.00-0.01)	0.01 (0.00-0.01)	0.177	0.00 (0.00-0.01)	0.01 (0.00-0.01)	0.693
AMR	226 ± 76	244 ± 74	0.106	229 ± 85	259 ± 69	0.052	221 ± 63	217 ± 76	0.804
% DS in stent	14.0 (8.0-18.2)	12.0 (8.8-17.2)	0.654	14.0 (7.0-18.0)	12.0 (8.8-17.2)	0.871	15.0 (8.0-21.0)	13.0 (7.5-17.5)	0.429
% DS <20%	68 (76.4)	70 (78.7)	0.857	37 (75.5)	46 (80.7)	0.682	31 (77.5)	24 (75.0)	1.000
SAQ scores									
Summary	84.2 ± 11.2	85.4 ± 11.0	0.484	82.5 ± 12.7	86.0 ± 10.2	0.113	86.5 ± 8.1	84.1 ± 12.5	0.342
Subdomains									
Physical limitation^a^	85.4 ± 17.9	87.4 ± 18.0	0.472	84.8 ± 18.0	88.2 ± 17.7	0.341	86.2 ± 18.0	85.8 ± 18.9	0.941
Stability^a^	74.4 ± 25.6	70.1 ± 27.8	0.316	72.7 ± 25.2	70.0 ± 27.8	0.614	76.52 ± 26.5	70.2 ± 28.3	0.381
Frequency^a^	96.1 ± 9.9	97.9 ± 6.1	0.154	94.8 ± 11.7	99.2 ± 3.3	0.007	97.8 ± 6.7	95.2 ± 9.1	0.176
Freedom from angina^b^	72 (82.8)	77 (86.5)	0.629	39 (78.0)	56 (93.3)	0.040	33 (89.2)	21 (72.4)	0.687
Satisfaction^a^	85.7 ± 13.2	85.9 ± 11.5	0.919	84.1 ± 15.1	86.2 ± 11.7	0.430	87.8 ± 10.0	85.3 ± 11.1	0.342
Perception^a^	70.3 ± 17.1	69.7 ± 18.4	0.838	67.8 ± 18.0	69.9 ± 18.1	0.560	73.9 ± 15.2	69.4 ± 19.2	0.315
SYNTAX score									
Functional	6.0 (5.0-8.0)	6.0 (3.0-8.0)	0.961	6.0 (3.0-7.0)	6.0 (2.0-7.0)	0.451	6.0 (5.0-8.0)	7.0 (5.0-9.0)	0.423
Residual functional	0.0 (0.0-4.0)	0.0 (0.0-3.0)	0.390	0.0 (0.0-0.0)	0.0 (0.0-0.0)	0.744	5.0 (2.0-6.0)	5.0 (2.5-7.0)	0.343

Values are mean ± SD, median (Q1-Q3), or n (%).

AMR = angiography-derived microvascular resistance; DS = diameter stenosis; μFR = Murray’s law–based quantitative flow ratio; PPGI = pullback pressure gradient index; SAQ = Seattle Angina Questionnaire. Other abbreviations as in Table 1.

a The number of patients providing responses for each SAQ subdomain was as follows: Physical Limitation score (N = 168), Stability score (N = 158), Frequency score (N = 176), Satisfaction score (N = 174), and Perception score (N = 165).

b Score = 100.

Within the AOF subgroup, although the SAQ Summary, Physical Limitation, Stability, Satisfaction, and Perception scores were comparable between lesion phenotypes, the angina frequency scores were significantly lower in patients with diffuse disease compared with those with focal disease (94.8 ± 11.7 vs 99.2 ± 3.3; *P* = 0.007), and the rate of freedom from angina was 78.0% (39 of 50; 95% CI: 64.8-87.2) versus 93.3% (56 of 60; 95% CI: 83.0-97.2; *P* = 0.040).

**Figure 1:**
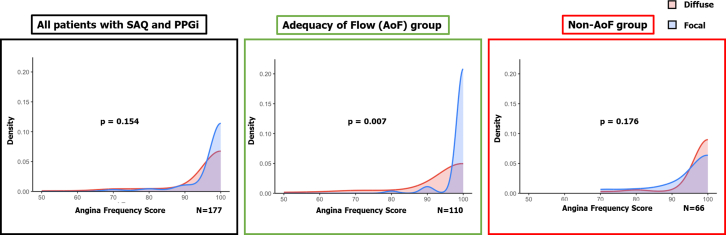
Angina Frequency Score by Lesion Phenotype and Flow Adequacy This figure shows the distribution of Seattle Angina Questionnaire Angina Frequency scores by physiological lesion phenotype and postprocedural adequacy of flow. Density curves are presented for the overall cohort, patients achieving adequacy of flow, and patients without adequacy of flow. Among patients achieving adequacy of flow, diffuse phenotype was associated with greater residual angina, whereas no clear separation was observed in patients without adequacy of flow. AoF = adequacy of flow; PPGi = pressure pullback gradient index; SAQ = Seattle Angina Questionnaire.

Figure 1 separately illustrates distribution curves of the angina frequency score for the overall cohort as well as the AOF and non-AOF subgroups (distributions of SAQ Summary and subscores are presented in Supplemental Figure 2). The detailed distributions of SAQ Angina Frequency scores and categories according to adequacy of flow and lesion phenotype are presented in Supplemental Figures 3 and 4.

### Distribution of PPGI by Angina Status

**Figure 2:**
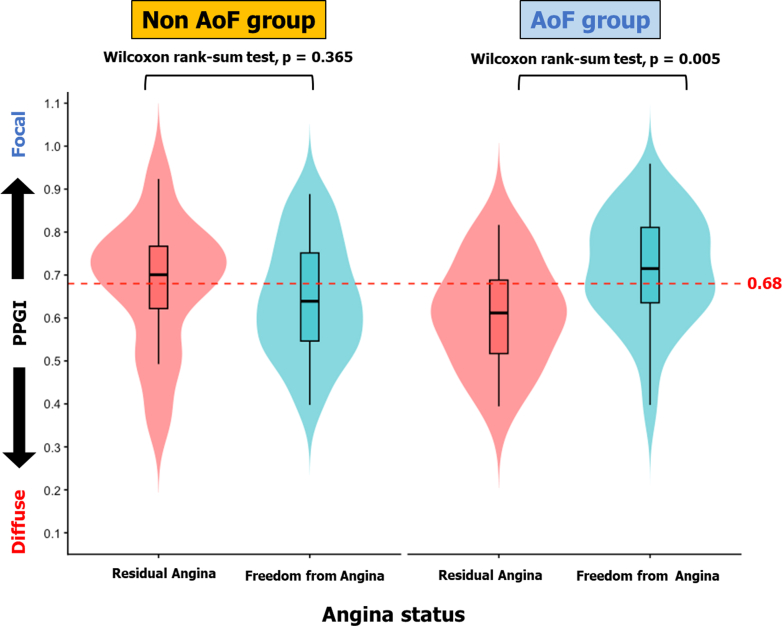
Angina Status as a Function of Adequacy of Flow and the Impact of the Pressure Pullback Gradient Index Violin plots depict the distribution of treated-vessel pressure pullback gradient index according to angina status and postprocedural adequacy of flow. Median values and quartile 1 to quartile 3 ranges are shown. Patients with residual angina are indicated in orange and angina-free patients in green. The dashed red line denotes the cohort median pressure pullback gradient index (0.68). *P* values were derived from 2-sided Wilcoxon rank-sum tests. Abbreviations as in Figure 1.

Table 3 shows the number of patients with residual angina and those free from angina, along with their median PPGI values, stratified according to AOF category. The median PPGI (0.68) in patients with functional non-AOF did not differ significantly between patients with or without residual angina at follow-up (0.70; Q1-Q3: 0.62-0.72 vs 0.64; Q1-Q3: 0.55-0.75; *P* = 0.364). By contrast, patients with AOF and residual angina had a significantly lower PPGI value than those who were free from angina (0.61; IQR 0.52-0.69 vs 0.72; Q1-Q3: 0.64-0.81; *P* = 0.005). Figure 2 shows the box plots of baseline PPGI according to angina status. Spline analyses of the relationship between PPGI and angina frequency are shown in Supplemental Figure 5, demonstrating a modest positive association in patients with AOF, whereas no consistent relationship was observed in those without AOF.

**Table 3:** Angina Status Stratified by Achievement of Adequacy of Flow

	Angina at 2 Years		Freedom From Angina		*P* Value
	No. of Patients	PPGI	No. of Patients	PPGI	
Non-AOF group	12	0.70 (0.62-0.72)	54	0.64 (0.55-0.75)	0.364
AOF Group	15	0.61 (0.52-0.69)	95	0.72 (0.64-0.81)	0.005

Values are presented as patient counts (n) and median (Q1-Q3). *P* values indicate statistical significance in median completeness rates between angina at 2 years and no angina groups within each completeness category (residual functional SYNTAX score = 0 and residual functional SYNTAX score >0).

PPGI = pullback pressure gradient index. Other abbreviations as in Table 1.

## Discussion

The main findings of the present study are as follows:

• In a CCS population with low anatomical complexity (median anatomical SYNTAX score: 7.0; Q1-Q3: 5.0-11.0), only 61% of patients achieved global AOF after PCI, as per a retrospective core lab functional assessment using angiography and including all vessels with a diameter of ≥ 1.5 mm.
• Patients with global AOF and a diffuse disease phenotype had worse SAQ scores at follow-up compared with those with a focal phenotype.
• The pre-PCI PPGI values among patients who failed to achieve functional AOF were not significantly different when comparing those with or without angina at follow-up.

Before the recent advances in angiography-derived physiological assessment, it was neither practical nor feasible to perform a systematic hemodynamic evaluation of all relevant vessels, including well-sized side branches, using wire-based FFR before and after PCI. This limitation hindered the assessment of the completeness of functional revascularization using validated scores such as the residual functional SYNTAX score, as well as establishing the optimal hemodynamics in treated vessels after PCI. In the present study, the use of μFR overcame these constraints, enabling a comprehensive physiological assessment and allowing the functional pattern of disease to be evaluated in the context of complete and optimal revascularization and without residual ischemic stenoses.

The rate of functional AOF was only 61% despite a low median anatomical SYNTAX score of 7.0 and universal use of intravascular imaging.^24^ Among patients who achieved AOF, those with a diffuse physiological disease pattern had lower SAQ scores compared with those with a focal pattern. This comparison was restricted to the AOF subgroup, and no direct comparison of diffuse versus focal disease patterns was performed across patients with and without AOF. Therefore, failing to achieve AOF, defined as the presence of residual functionally significant untreated lesions and/or suboptimal functional results in treated lesions, might influence the impact of the baseline anatomical disease phenotype in predicting post-PCI symptom relief.

The disease phenotype might be weighted against the combination of residual ischemia and suboptimal post-PCI functional results to more accurately discriminate the risk of having residual/recurrent symptoms. Although our findings do not replicate the strong phenotype-related associations reported in previous studies, they reaffirm those observations within the context of adequate treatment.^8^ Our study confirms worse post-PCI functional results in patients with a physiologically diffuse versus focal lesion phenotype (Table 2).^6^ It represents a step forward in the understanding of global post-PCI functional evaluation, enabled by using advanced angiography-derived physiological measures.

Even when physiological results after PCI appeared optimal, the presence of underlying diffuse disease was associated with a higher likelihood of residual or recurrent symptoms. This finding suggests that additional mechanisms may contribute to the higher rates of residual or recurrent angina observed in patients with a diffuse disease phenotype, despite the global adequacy of flow after PCI. The coexistence of different pathophysiological mechanisms and potential treatment targets, such as microvascular dysfunction and vasospasm, in this patient subset should be taken into consideration and explored. Moreover, despite achieving optimal functional results, patients with diffuse disease baseline may also have an increased risk of rapid disease progression or in-stent restenosis compared with patients with focal disease, both of which can contribute to the recurrence of angina.

### Study Limitations

First, despite the fact that μFR was validated against wire-based FFR, the sensitivity of PPGI based on μFR has not yet been validated with FFR pullback. Second, the coexistence of microvascular disease or vasomotor disorders, which can significantly contribute to angina status, has not been evaluated and represents a critical gap in knowledge that needs to be addressed in future studies.

Third, the results of this study should be considered as descriptive because the reduced sample size limits multivariable adjustment. Fourth, due to the lack of baseline SAQ assessment, it remains uncertain whether the presence of angina after the index procedure reflected preexisting pathology (such as microvascular dysfunction, vasospasm, or incomplete revascularization) or represented newly developed symptoms related to disease progression over time. Fifth, baseline SAQ data were not collected in ASET Japan. Therefore, angina status assessed at 2 years reflects the combined influence of baseline symptoms, postprocedural physiology, and disease progression during follow-up and residual, recurrent, and de novo angina after PCI cannot be distinguished.

In addition, this is a post hoc analysis, and the data presented are hypothesis generating. Nevertheless, this study could be the foundation of a comprehensive and prospective observational registry. Sixth, The ASET-Japan trial was originally designed with protocol-defined follow-up and systematic collection of concomitant medication data up to 4 months. Concomitant medication data were collected during this period, and concomitant medication use up to 3 months is summarized in Supplemental Tables 1 and 2.

Seventh, the μFR threshold of 0.90 is based on outcome-driven evidence from prior studies and does not represent a universally validated physiological standard for angina relief (Supplemental Tables 2 and 3). Finally, the mechanisms of ischemia with nonobstructive coronary arteries, including coronary microvascular dysfunction and vasomotor disorders, were not evaluated in this study, which limits mechanistic interpretation of angina persistence after PCI.

## Conclusions

Among patients who achieved AOF, those with a focal physiological disease pattern experienced more favorable angina outcomes at 2 years compared with those with diffuse disease. These findings suggest that evaluating the long-term status of angina after PCI requires assessment of not only the pre-PCI pathophysiological pattern of CAD (diffuseness) in the treated vessel but also the AOF.

## Funding Support and Author Disclosures

The ASET-Japan study is an investigator-initiated study supported by a grant provided by Boston Scientific. The study sponsor was Meditrix Research organization. The grant giver was not involved in the study design, study operation or in the writing of the manuscript.

Dr Miyashita has received a research grant from OrbusNeich KK. Dr Bianchini has received fees from Medtronic for conducting educational events. Dr Muramatsu has received honoraria from Boston Scientific and Daiichi-Sankyo. Dr Nakazawa has received honoraria from Abbott Medical, Boston Scientific, Terumo Corp., HeartFlow Japan, Daiichi-Sankyo, OrbusNeich, Amgen, Shockwave Medical Japan, and Medtronic Japan. Dr Kozuma has received research funds from Abbott Medical and honoraria for lectures from Abbott Medical, Medtronic, Shockwave Medical, and Boston Scientific Japan. Dr Morino has received an unrestricted research grant and honoraria from Boston Scientific Japan and Daiichi-Sankyo. Dr Nakamura has received grants from Daiichi-Sankyo, honoraria from Boston Scientific Japan, and an endowed course supported by Boston Scientific Japan. Dr Garg has received consultant fees from Biosensors and honoraria from Novartis. Dr Tanabe has received honoraria for lectures from Boston Scientific, Abbott Medical, HeartFlow, Medis, and Daiichi-Sankyo. Dr Spertus has received institutional grants from Imbria, Janssen, ACCF, and Lexicon, and license fees for copyright of SAQ, KCCQ, and PAQ, as well as consultant fees from Imbria, VentricHealth, BMS, Cytokinetics, Janssen, and Terumo. Dr Serruys has served as a consultant for Sahajanand Medical Technologies, Meril Life Sciences, Philips, and Xeltis, outside the submitted work. All other authors have reported that they have no relationships relevant to the contents of this paper to disclose.
