# Supplementary material for: Impact of Angiography-Derived Physiological Patterns of CAD and Optimal Hemodynamics Post-PCI on Residual Angina
Source: JACC Asia. 2026 May 16;6(7):1108–18. doi: 10.1016/j.jacasi.2026.03.023 (PMC13350794; doi:10.1016/j.jacasi.2026.03.023)
Supplement: Supplemental Material [file mmc1.docx]

**Supplementary file**

**Supplemental Methods**

In logistic regression univariate analyses, the increase of continuous value of PPGi (more focal disease) had Odds Ratio of 1.44 (95%CI: 0.96 - 2.17), whereas categorical variable of physiologically diffuse CAD (PPGi ≤ 0.68) in treated vessel had Odds ratio of 0.234 (95%CI: 0.078-0.702). Odds ratio of AoF was 1.319 (95%CI: 0.581 -2.995). These associations did not reach statistical significance.

A multivariable logistic regression analysis demonstrated that the interaction between diffuse CAD and AoF is significant (OR 0.098, 95%CI [0.016 – 0.584], p<0.01). After adjusting the interaction, the ORs of diffuse phenotype and AoF are 24.574 (95%CI: 1.187 - 508.909), and 32.033 (95%CI: 2.422 - 423.626) respectively. Diffuse phenotype became a predictor rather than a protector for no angina status at 2 years.

The spline plots of PPGi and Angina Frequency score are shown in the Supplementary Figure 5. For the patient with AoF (green line), when PPGi is below 0.7, there appears a linear relationship between PPGi and Angina Frequency, whereas with PPGi greater than 0.7 the line becomes flat at the level of angina frequency of 100. In contrast, in patients without AoF, the line fluctuates over the various PPGi values.

Considering this fluctuation of the odds ratio, a significant interaction between AoF and PPGi, and spline plots, the patients were stratified according to the status of AoF.

In a univariate logistic regression analysis using this alternative cut-point (Tertile), the diffuse phenotype was associated with angina-free status at 2 years (OR 3.92, 95% CI 1.30 -11.89; p = 0.016). In the multivariate logistic regression analysis with continuous PPGI, AoF and the interaction between the two variables, Odds ratio were 0.264 (95%CI 0.07 - 1.06), 0.655 (0.28 - 1.56) and 2.950 (1.24 - 6.99).

**Supplementary Table 1. Comparison of Seattle Angina Questionnaire Scores Between AoF and Non-AoF**

|  |  | **AoF group (N=111)** | **Non AoF group  (N=66)** | **p value** |
| --- | --- | --- | --- | --- |
| **SAQ Summary score** | | 84.4 (8.5) | 85.5 (10.3) | 0.524 |
| **SAQ subdomain** | |  |  |  |
|  | **Physical limitation score*** | 86.6 (17.8) | 86.0 (18.2) | 0.841 |
|  | **Stability score*** | 71.2 (26.6) | 73.7 (27.2) | 0.572 |
|  | **Frequency score*** | 97.2 (8.5) | 96.7 (7.9) | 0.687 |
|  | **Freedom from angina (score = 100)** | 95 (86.4%) | 54 (81.8%) | 0.652 |
|  | **Satisfaction score*** | 85.2 (13.3) | 86.7 (10.5) | 0.406 |
|  | **Perception score*** | 68.9 (18.0) | 71.9 (17.1) | 0.278 |

Values are presented as mean (standard deviation) or number of patients (%), as appropriate. SAQ indicates Seattle Angina Questionnaire; AoF, adequacy of flow.

**Supplementary Table 2. Concomitant Medication Use at Baseline, Discharge, and 3 Months**

| **Medication** | **Baseline** | **Discharge** | **3-month** |
| --- | --- | --- | --- |
| **Statin** | 171 (91.9%) | 171 (91.9%) | 171 (91.9%) |
| **Ezetimibe** | 52 (28.0%) | 62 (33.3%) | 62 (33.3%) |
| **PCSK9 inhibitor** | 0 (0.0%) | 0 (0.0%) | 0 (0.0%) |
| **PPI** | 142 (76.3%) | 142 (76.3%) | 142 (76.3%) |
| **β-blocker** | 17 (9.1%) | 17 (9.1%) | 17 (9.1%) |
| **CCB** | 11 (5.9%) | 14 (7.5%) | 14 (7.5%) |
| **ACEi** | 3 (1.6%) | 4 (2.2%) | 4 (2.2%) |
| **ARB** | 7 (3.8%) | 9 (4.8%) | 9 (4.8%) |
| **Nitrates** | 7 (3.8%) | 8 (4.3%) | 8 (4.3%) |

Data are presented as number of patients (%). ACEi, angiotensin-converting enzyme inhibitor; ARB, angiotensin receptor blocker; CCB, calcium channel blocker; PCSK9, proprotein convertase subtilisin/Kexin type 9; PPI, proton pump inhibitor.

**Supplementary Table 3. Post-PCI Physiological Targets in Prior Clinical Trials**

| **Trial** | **Physiological index** | **Post-PCI target definition** | **Purpose of threshold** |
| --- | --- | --- | --- |
| FLAVOUR I | Wire-based FFR | FFR ≥ 0.88 | Definition of successful PCI |
| FLAVOUR II | Angiography-derived FFR (QFR/μFR) | QFR ≥ 0.88 | Definition of optimal PCI |
| TARGET-FFR | Wire-based FFR | FFR ≥ 0.90 | Prognostically favorable post-PCI physiology |
| FAVOR III (China / Europe) | Angiography-derived FFR (QFR) | QFR ≥ 0.88 | Outcome-driven PCI guidance |
| PANDA III | Angiography-derived physiology (QFR) | QFR ≥ 0.80 | Consistency between anatomy and physiology |

This table summarizes post–percutaneous coronary intervention (PCI) physiological target thresholds used in prior clinical trials and their intended purpose. FFR indicates fractional flow reserve; QFR, quantitative flow ratio; μFR, angiography-derived fractional flow reserve.

**Supplementary Figure 1: Flowchart of functional assessment**

This flowchart illustrates the lesion-level and patient-level analyses performed in the ASET-Japan study. Investigators’ initial intention-to-treat decisions were compared with retrospective CoreLab reassessment using quantitative coronary angiography (QCA) and angiography-derived μFR (cut-off ≤0.80). Actual treatment and subsequent device success were adjudicated by the CoreLab, where device success was defined as post-PCI QCA diameter stenosis <20% and ΔμFR <0.05. Lesions and patients were stratified according to treatment, device success, and physiological results. At the patient level, 186 subjects were analyzed, of whom 144 (151 lesions) achieved device success and 42 (42 lesions) did not, with corresponding Seattle Angina Questionnaire (SAQ) scores shown.

**Supplementary Figure 2: Distribution of SAQ-AF categories by lesion phenotype**

Bar graphs illustrate the proportion of patients with daily/weekly angina (SAQ-AF <60), monthly angina (60–99), and no angina (=100) according to baseline lesion phenotype (diffuse, red; focal, blue). Results are shown for the overall cohort, as well as stratified by achievement of global adequacy of flow (AoF vs. non-AoF). In the overall population, there was no significant difference between diffuse and focal disease (p=0.552). Among patients with AoF, there was a trend toward a higher proportion of patients without angina in the focal group (p=0.056), whereas no significant difference was observed in the non-AoF group (p=0.153).

**Supplementary Figure 3: Density plots of SAQ domain scores by focal lesion status, stratified by completeness of revascularisation.**

Density plot curves comparing patients with focal versus diffuse lesions for each SAQ domain: Summary Score, Physical Limitation, Stability, Angina Frequency, Satisfaction, and QOL.

Panels show distributions in (A) the overall cohort, (B) the AoF group, and (C) the Non-AoF group based on residual Functional SYNTAX score.

**Supplementary Figure 4:** **Distribution of SAQ-AF score stratified by AoF and lesion phenotype**

Density plots of Seattle Angina Questionnaire Angina Frequency (SAQ-AF) scores stratified by adequacy of flow (AoF, green) versus non-AoF (red). P-values are derived from Wilcoxon rank-sum tests comparing AoF and non-AoF groups within each panel.

**Supplementary Figure 5: Restricted Cubic Spline Analysis of PPGI and Angina Frequency**


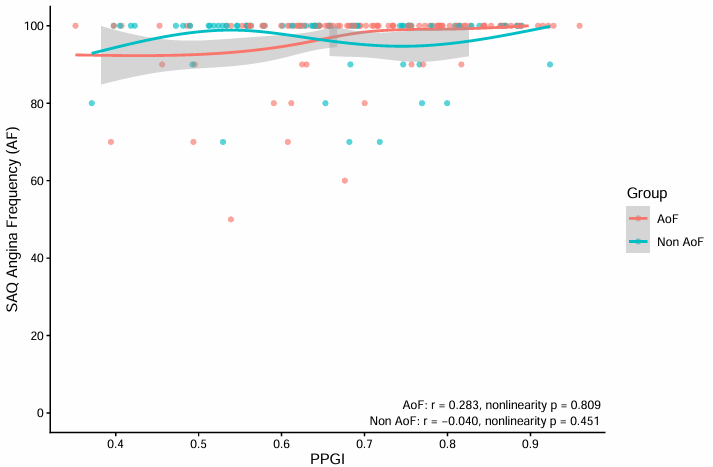


This figure illustrates the relationship between continuous pressure pullback gradient index (PPGI) and Seattle Angina Questionnaire Angina Frequency (SAQ-AF) scores at 2 years, stratified by postprocedural adequacy of flow (AoF). Red points and curves represent patients achieving AoF, whereas blue points and curves represent patients without AoF. Among patients achieving AoF, higher PPGGI showed a modest positive association with angina frequency, while no consistent relationship was observed in patients without AoF. Tests for nonlinearity were not significant in either group.
AoF = adequacy of flow; PPGI = pressure pullback gradient index; SAQ-AF = Seattle Angina Questionnaire Angina Frequency.
